# Cognitive-Processing Bias in Chinese Student Teachers with Strong and Weak Professional Identity

**DOI:** 10.3389/fpsyg.2017.00784

**Published:** 2017-05-15

**Authors:** Xin-qiang Wang, Jun-cheng Zhu, Lu Liu, Xiang-yu Chen

**Affiliations:** School of Psychology, Jiangxi Key Laboratory of Psychology and Cognition Science, Center for Mental Health Education and Research, Jiangxi Normal UniversityNanchang, China

**Keywords:** student teachers, professional identity, cognitive-processing bias, coding bias, recognition bias

## Abstract

Professional identity plays an important role in career development. Although many studies have examined professional identity, differences in cognitive-processing biases between Chinese student teachers with strong and weak professional identity are poorly understood. The current study adopted Tversky’s social-cognitive experimental paradigm to explore cognitive-processing biases in Chinese student teachers with strong and weak professional identity. Experiment 1 showed that participants with strong professional identity exhibited stronger positive-coding bias toward positive profession-related life events, relative to that observed in those with weak professional identity. Experiment 2 showed that participants with strong professional identity exhibited greater recognition bias for previously read items, relative to that observed in those with weak professional identity. Overall, the results suggested that participants with strong professional identity exhibited greater positive cognitive-processing bias relative to that observed in those with weak professional identity.

## Introduction

Teachers’ professional identity has recently become an independent research topic in the field of education (Beijaard et al., 2004). Moreover, as student teachers are the main teaching force of the future, their professional identity has also attracted increasing attention in research (Wang et al., 2010; Trent, 2011; Izadinia, 2013; Ma et al., 2013; Stenberg et al., 2014). Some studies have shown that student teachers with strong professional identity promoted their professional skills and personal development and exhibited positive attitudes toward educational contexts (Beijaard et al., 2004; Zhang, 2006; Ma et al., 2013).

Student teachers’ professional identity is dynamic and develops during the process involved in learning to teach (Hong, 2010; Trent, 2011; Izadinia, 2013). In other words, numerous factors, such as motivation and previous experiences, influence the development of student teachers’ professional identity (Findlay, 2006; Olsen, 2008; Rodgers and Scott, 2008). Current research examining student teachers’ professional identity is in the preliminary stage (Feng et al., 2010; Timoštšuk and Ugaste, 2010) and tends to involve theoretical discussion and scale measurement rather than empirical studies (Timoštšuk and Ugaste, 2010; Wang et al., 2011; Lei et al., 2012). For example, a survey examining student teachers’ professional identity via questionnaires showed that professional cognition and emotion were the most important components of professional identity (Wang et al., 2010). In another study, student teachers with strong professional identity were able to overcome harsh environments and promote their professional development (Walkington, 2005; Song and Wei, 2006). In addition, Cai and Liu (2010) conducted field research to examine teachers’ professional identity and found that they were at the core of self-identity. Specifically, the strength of student teachers’ professional identity was positively associated with their enthusiasm for teaching and negatively associated with burnout and intention to resign (Luo et al., 2014). Moreover, student teachers with strong professional identity exhibited higher levels of satisfaction with life and lower levels of anxiety regarding the future, relative to those with weak professional identity (Wang et al., 2011). In summary, the above-mentioned studies showed that student teachers’ professional identity could influence their status and the quality of their professional lives (Cai and Liu, 2010).

Furthmore, some studies have shown that teachers with strong and weak professional identity exhibited different types of cognitive-processing bias toward profession-related life events (Wei, 2010; Kou and Zhang, 2013). For instance, the primary and middle school teachers who were experiencing professional-identity crisis exhibited stronger negative coding bias and recognition bias. In other words, teachers who were experiencing professional-identity crisis exhibited cognitive-processing bias toward negative profession-related life events, which resulted from negative professional self-schemas (Kou and Zhang, 2013). Some researchers have posited that self-schemas affected category accessibility in social-information processing (Kuiper, 1981; Kuiper and Derry, 1981; Bargh, 1982). In other words, self-schemas were based on previous experience and stored memories (Markus, 1977), which affected individuals’ cognitive-processing biases toward self-relevant information. Therefore, self-schemas play a crucial role in guiding student teachers with both strong and weak professional identity in the selection and processing of consistent profession-related life events (Sanitioso et al., 1990). Therefore, Wei (2008) adopted Tversky’s social-cognitive experimental paradigm (Tversky and Marsh, 2000) to compare the characteristics of social-cognitive processing of profession-related life events between teachers with strong and weak professional identity. The results showed that teachers with strong professional identity exhibited positive cognitive-processing bias, while those with weak professional identity exhibited negative cognitive-processing bias involving coding and recognition.

**FIGURE 1:**
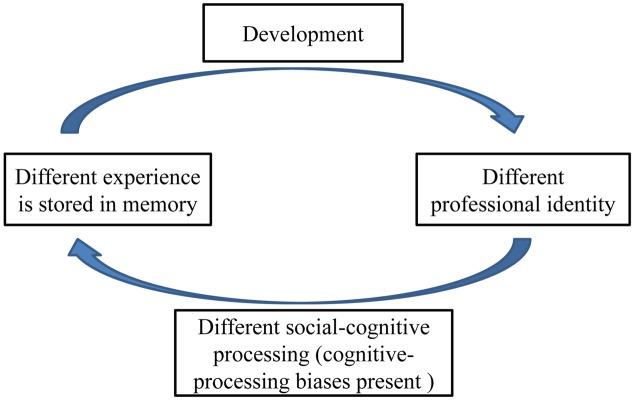
**The reciprocal influence between student teachers’ professional identity and cognitive processing of profession-related life events**.

Previous research has focused on cognitive-processing bias involving teachers’ profession-related life events in teachers with both strong and weak professional identity (Wei, 2008; Kou and Zhang, 2013). In addition, some studies have shown that teachers’ professional identity developed during training (Sutherland et al., 2010; Wang and Zhang, 2012; Izadinia, 2013). Therefore, training could play an important role in the development of student teachers’ professional identity (Wang and Zhang, 2012). Professional identity in student teachers has been defined as their perception and experience of their identity, which are expected to affect their professional lives in the future. A number of studies have shown that student teachers professional identity could change as they experience different life events during their teaching careers (Wei, 2008; Kou and Zhang, 2013). Based on the results of the above-mentioned studies (Wei, 2008), cognitive processing of profession-related life events could develop differently between student teachers with strong and weak professional identity, and this could affect their perception and experience of professional-related life events that occur during their teaching careers. In addition, the subsequent experience of different life events could restructure their professional identity, and this could affect their cognitive processes, which could influence their perception and experience further (see **Figure 1**). However, there is no empirical evidence indicating differences in cognitive-processing biases involving profession-related life events between student teachers with strong and weak professional identity. Therefore, the current study adopted Tversky’s social-cognitive experimental paradigm (Tversky and Marsh, 2000), to compare cognitive-processing biases between student teachers with strong and weak professional identity. If cognitive-processing bias plays a vital role in the development of student teachers’ professional identity, it would require consideration as an important factor in teacher education programs designed to cultivate student teachers’ professional identity.

## Theoretical Framework

### Teachers’ Professional Identity

The last 20 years of research in educational psychology have shown that teachers’ professional identity constitutes an important research topic (Beijaard et al., 2004), and an increasing number of studies have focused on this issue (Izadinia, 2013). Some studies have shown that teachers’ professional identity was dynamic and changeable (Maclean and White, 2007; Beijaard et al., 2004; Beauchamp and Thomas, 2009; Izadinia, 2013). In addition, it was established and developed based on self-identity and social identity and affected by numerous factors. For instance, one study found that teachers’ professional identity was negatively associated with career burnout (Gaziel, 1995; Schepens et al., 2009). Similarly, Moore and Hofman (1988) reported that, even in harsh working environments, strong professional identity could reduce the intention to resign effectively.

It is clear that teachers’ professional identity is established during training (Wang and Zhang, 2012). In student teachers, the development of professional identity was shown to be a vital factor in their qualification as teachers and exerted a strong effect on their learning and work (Izadinia, 2015). Moreover, numerous studies have shown that student teachers’ professional identity was affected by various types of information in teacher evaluations (Putnam and Borko, 1997; Wideen et al., 1998). The above-mentioned results indicate that student teachers’ professional identity could affect their cognitive processing.

### Cognitive-Processing Bias in Professional Identity

Cognitive-processing bias theory suggests individuals could develop cognitive-processing biases involving self-related information (Rusting and Larsen, 1998). Cognitive-processing bias has been observed in perception, attention, and memory tasks (Heikkilä et al., 2012; Yu et al., 2015). In addition, one study found that external environments and personality traits were influenced by cognitive-processing bias (Rusting and Larsen, 1998). Other studies examined cognitive-processing bias in different groups, such as teachers (Zhao et al., 2011), middle school students with depressive symptoms (Yu et al., 2015) and Internet addicts (Wang, 2008), using Tversky’s social-cognitive experimental paradigm. The results showed that individuals were particularly sensitive to information that was consistent with their self-schema (Sanitioso et al., 1990). For instance, teachers experiencing professional-identity crisis exhibited stronger negative cognitive-processing bias involving career-related life events (Zhao et al., 2011), relative to those observed in teacher who were not experiencing professional-identity crisis, and therefore showed characteristics of negative social-cognitive processing bias in coding and recognition. However, the ways in which teachers’ professional identity affects cognitive-processing bias in student teachers remain unclear.

## Experiment 1

### Participants

Three hundred student teachers completed the Student Teachers’ Professional Identity Scale developed by Wang et al. (2010). In accordance with the methods used in a previous study (Li et al., 2011), we classified the scores as high or low according to the sequential scores; high scores (*M*_total_ + 1 *SD*_total_) indicated strong professional identity (Strong Group) and low scores (*M*_total_ - 1 *SD*_total_) indicated weak personal identity (Weak Group). Following this process, 22 participants with a total scores above 48 (*M* = 51.82, *SD* = 2.91) were assigned to the groups with strong professional identity, and 23 participants with total scores below 41 (*M* = 37.78, *SD* = 3.01) were assigned to the groups with weak professional identity. Professional identity scores have significant difference between the two groups, *t*(43) = 15.89, *p* < 0.001, Cohen’s *d* = 4.85. Of the 45 participants (*M* = 21.04, *SD* = 1.22), 16 were men and 29 were women.

### Design

The study included a two-factor mixed study design, with professional identity (strong and weak) as the between-subjects variable, profession-related life events (positive, neutral, and negative) as the within-subjects variable, and the coding bias of sentence type as dependent variables.

### Procedure

Participants were assessed separately. The experiment included two stages: the learning stage and the coding-test stage.

#### Learning Stage

Each participant was provided with reading material and the following instructions: “Please read the material carefully for 5 min. We will test your knowledge of the detailed content of the material.” After reading the material for 5 min, participants were required to count backward from 500 in threes for 3 min. The researcher then retrieved the reading material and distributed the coding material.

#### Coding-Test Stage

Participants received the following instructions: “You will receive reading material that is identical to the material you read previously. Please code the sentences followed by parentheses. Please insert ‘√’ to signify positivity, happiness, or relaxation; ‘X’ to signify negativity, worry, or depression; and ‘O’ to signify description or objectivity according to your own understanding and experience of the words. You have 10 min to complete the test.”

### Materials

#### Professional Identification Scale

The scale, which was used in previous studies involving Chinese participants, was used to measures student teachers’ professional identity (Wang et al., 2010; Zhang et al., 2011). The scale includes 12 items divided between the following four dimensions: professional willingness, which represents the respondent’s expectations and preparedness for a teaching career (three items; e.g., “I am willing to communicate with pupil”); professional volition, which represents the respondent’s willingness to continue a teaching career when faced with other career options (three items; e.g., “I’ll be a teacher for life”); professional values, which focuses mainly on value judgments regarding the teaching profession (three items; e.g., “I think student teachers are respected”); and professional efficiency, which focuses mainly on self-efficacy regarding the teaching profession (three items; e.g., “I have the ability to master teaching skills”). Responses are provided using a Likert scale ranging from 1 (*strongly disagree*) to 5 (*strongly agree*). Total scores range from 12 to 60, and higher scores indicate stronger professional identity. The Cronbach’s α for the scale was 0.86, the test–retest reliability coefficient for the scale was 0.91, and the criterion validity coefficient for the scale was 0.84 (Zhang et al., 2011). The correlations between scale items and total scores are presented in **Table 1**.

**Table 1:** Correlations between total scores and items of the Student Teacher Professional Identity Scale.

	Items	Correlation with the total score	Dimensions
6	I hope to aid the development of healthy personalities in students	0.53^∗∗^	a
7	I am willing to communicate with excellent teachers	0.69^∗∗^	a
11	I am willing to communicate with pupils	0.62^∗∗^	a
2	If I could choose another career, I would still choose to be a teacher	0.67^∗∗^	b
4	I will be a teacher for life	0.67^∗∗^	b
12Δ	After working many years, I may engage in other types of work	0.71^∗∗^	b
1	I think that student teachers are respected	0.51^∗∗^	c
8	I think that teachers’ social status is high	0.54^∗∗^	c
10	I think the teachers’ work is respected by others	0.51^∗∗^	c
3	I think I can become a qualified teacher	0.74^∗∗^	d
5	I think I can become an excellent teacher	0.71^∗∗^	d
9	I have the ability to master teaching skills	0.59^∗∗^	d


*Dimensions: a = professional willingness; b = professional volition; c = professional values; d = professional efficiency; ^∗∗^*p* < 0.01; Δ = reverse-scored item.*

#### Reading Material

The reading material used in the study was “The Story of Two Colleagues” (see Appendix A), which was created by Wei (2008). The story refers to the participant as “you” and describes profession-related life events involving two colleagues (e.g., “You are a teacher at a middle school and have two colleagues named Zhang and Li”). The story includes 54 sentences describing profession-related life events involving topics such as teaching or salary (18 positive, 18 neutral, and 18 negative).

In addition, the α coefficients for homogeneity reliability for the three sentence types were 0.80 for positive items, 0.71 for neutral items, and 0.81 for negative items, indicating that the material fulfilled psychometric requirements.

#### Coding Material

The coding material was identical to the reading material (see Appendix A) but included parentheses at the end of some statements, to identify sentences that participants were required to encode [e.g., “The office was full of garbage that Zhang left behind ()”]. Participants were required to indicate whether the sentences were positive, neutral, or negative.

### Results

The results of tests of normality showed significant Skewness (*p* < 0.05), indicating that the data were not normally distributed, and the results of the test of homogeneity of variance showed no significant homogeneity (*p* > 0.05). Therefore, we performed a Mann–Whitney *U* test. The coding results for positive, neutral, and negative items for student teachers with strong and weak professional identity are shown in **Table 2**.

**Table 2:** Coding results for positive, neutral, and negative items for student teachers with strong and weak professional identity.

Sentence item type	Coding item type	Strong professional identity (*n* = 22)		Weak professional identity (*n* = 23)	
			
		*M*	*SD*	*M*	*SD*
Positive	Positive	16.91	2.00	14.91	3.45
	Neutral	0.91	2.00	2.35	2.90
	Negative	0.18	0.50	0.74	1.63
Neutral	Positive	3.73	3.80	5.57	3.73
	Neutral	12.59	4.80	10.09	4.74
	Negative	1.68	1.67	2.35	1.90
Negative	Positive	1.55	3.10	1.35	2.41
	Neutral	2.86	2.49	3.13	2.38
	Negative	13.59	3.78	13.52	3.46


**FIGURE 2:**
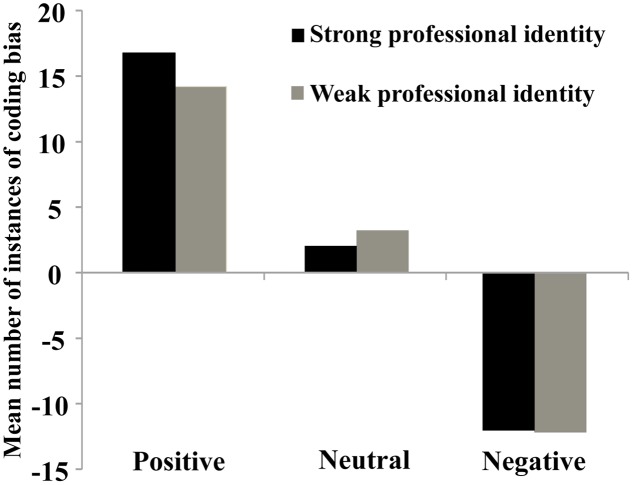
**Coding bias in student teachers with strong and weak professional identity**.

The formula for calculating the number of instances of positive coding bias was as follows: the number of instances of positive coding bias = the number of positively coded items minus the number of negatively coded items (Qian et al., 1998; Wei, 2008; Kou and Zhang, 2013). The results showed that the number of instances of positive coding bias involving positive items observed in individuals with strong professional identity was significantly higher relative to that observed for those with weak professional identity (*Z* = -2.32, *p* = 0.02). However, the numbers of instances of positive coding bias involving neutral (*Z* = -1.05, *p* = 0.30) and negative (*Z* = -0.13, *p* = 0.90) items did not have significant difference between individuals with strong and weak professional identity (**Figure 2**).

## Experiment 2

### Participants

Three hundred student teachers completed the Student Teachers’ Professional Identity Scale developed by Wang et al. (2010). The method used to assign participants to groups in Experiment 2 was identical to that described for Experiment 1. Twenty-one participants with total scores above 46 (*M* = 50.48, *SD* = 3.27) were assigned to the group with strong professional identity, and 19 participants with total scores below 42 (*M* = 38.05, *SD* = 5.09) were assigned to the group with weak professional identity. Professional identity scores did have significant difference between the two groups, *t*(38) = 9.27, *p* < 0.001, Cohen’s *d* = 3.01. Of the 40 participants (*M* = 20.48, *SD* = 1.30), 10 were men and 30 were women.

### Design

The study included a two-factor mixed design, with group (strong professional identity, weak professional identity) as the between-subjects variable, type of profession-related life event (original, additional) as the within-subjects variable, and recognition bias of sentence type as dependent variables.

### Procedure

Participants were assessed separately. The experiment included two stages: the learning stage and the recognition-test stage.

#### Learning Stage

The learning stage in Experiment 2 was identical to that described for Experiment 1, but the researcher distributed the recognition material, rather than the coding material, at the end of the stage.

#### Recognition-Test Stage

Participants received the following instructions: “Please answer the following 108 questions regarding the reading material. Please insert ‘√’ to signify consistency with the content of the reading material and ‘×’ to signify inconsistency with the content of the reading material. You have 15 min to complete the test.”

### Materials

#### Reading Material

The reading material for Experiment 2 (“The Story of Two Colleagues”) was identical to that described for Experiment 1.

#### Recognition-Test Material (See Appendix B)

The recognition test included 108 questions with the following unified format: colleague name (two colleagues) plus an activity or fact. There were 54 key sentences in the reading material, which included nine positive sentences, nine neutral sentences, and nine negative sentences depicting activities or behavior for each colleague. The test also included 54 additional sentences concerning the two colleagues (nine positive, nine neutral, and nine negative sentences for each colleague), which were not included in the original reading material but matched the sentence format.

### Results

The results of tests of normality showed no significant Skewness (*p* > 0.05), indicating that the data were normally distributed, and the results of the test of homogeneity of variance showed no significant homogeneity (*p* > 0.05). Therefore, we performed a 2 (group: strong professional identity, weak professional identity) × 2 (type of profession-related life event: original, additional) ANOVA. The numbers of correct recognition of positive, neutral, and negative items for participants with strong and weak professional identity are shown in **Table 3**.

**Table 3:** Results for correct recognition of positive, neutral, and negative items for student teachers with strong and weak professional identity.

Source of sentence item	Recognition item type	Strong professional identity (*n* = 21)		Weak professional identity (*n* = 19)	
			
		*M*	*SD*	*M*	*SD*
Original	Positive	17.14	1.06	14.58	2.01
	Neutral	14.90	1.73	14.53	1.93
	Negative	14.71	1.98	13.95	2.48
Additional	Positive	7.48	2.68	6.89	3.68
	Neutral	13.76	1.41	12.05	3.57
	Negative	13.67	1.43	11.32	3.07


Recognition bias was calculated using the difference in recognition between positive and negative items. The formula for calculating the number of instances of recognition bias was as follows: the number of instances of recognition bias = the number of correctly recognized positive items minus the number of correctly recognized negative items (Qian et al., 1998; Wei, 2008; Kou and Zhang, 2013). The results showed that student teachers from both groups were positively biased in the recognition of original items but negatively biased in the recognition of additional items (**Table 4**).

**Table 4:** Recognition bias in student teachers with strong and weak professional identity.

Recognition bias item type	Strong professional identity (*n* = 21)		Weak professional identity (*n* = 19)	
		
	*M*	*SD*	*M*	*SD*
Original	2.43	2.38	0.63	3.11
Additional	-6.19	2.58	-4.42	3.47


**FIGURE 3:**
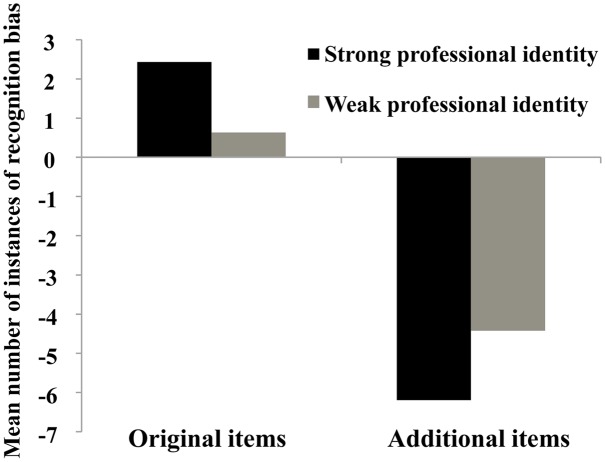
**Recognition bias in student teachers with strong and weak professional identity**.

The results of the ANOVA showed that the main effect of group was non-significant, *F*(1,38) = 0.001, *p* > 0.05. However, the main effect of type of profession-related life event, *F*(1,38) = 81.63, *p* < 0.001, $\eta_{p}^{2}$ = 0.68, and the interaction between group and type of profession-related life event were significant, *F*(1,38) = 5.56, *p* < 0.05, $\eta_{p}^{2}$ = 0.13. The results of simple effects analysis showed that participants with strong professional identity exhibited significantly greater recognition bias for original items relative to that observed in those with weak professional identity (*p* < 0.05). There was no significant difference in recognition bias between the two groups for the additional items (*p* = 0.07; **Figure 3**).

## Discussion

### Coding Bias in Student Teachers with Strong and Weak Professional Identity

The results of the study showed that coding bias differed between participants with strong and weak professional identity, which is consistent with previous findings (Wei, 2008; Kou and Zhang, 2013). Participants with strong professional identity coded positive sentence items as positive items, which showed a positive coding bias for positive sentence items. The results of Experiment 1 indicated that student teachers with strong professional identity exhibited coding bias in the cognitive processing of profession-related life events.

Why might student teachers with strong professional identity exhibit coding bias in cognitive processing? The results of Experiment 1 suggested that the strength of their professional identity was a contributory factor in this bias, in that it could have influenced the thresholds for activation of their professional self-schemas. In other words, the threshold for activation of positive professional self-schemas could have been lower, while that for activation of negative self-schemas could have been higher, for student teachers with strong professional identity, and this might have led to positive cognitive-processing bias (Wei et al., 2017). In addition, professional self-schemas could have been a contributory factor in this bias. Professional self-schemas are formed during learning via the observation of qualified teachers (Lamote and Engels, 2010). In addition, they undergo qualitative changes resulting from profession-related life events, which could lead to the development of cognitive-processing biases that are consistent with self-schemas (Kou and Zhang, 2013). When student teachers engaging in the learning process develop clear vocational goals and learn from qualified teachers, they could develop strong professional identity and positive professional self-schemas. Therefore, it is possible that student teachers with strong professional identity engage in selective processing and attentional bias concerning profession-related information, to maintain consistency between their cognitive-processing biases and professional self-schemas.

### Recognition Bias in Student Teachers with Strong and Weak Professional Identity

In the current study, participants with strong and weak professional identity exhibited differences in recognition bias, which is consistent with previous findings (Wei, 2008; Kou and Zhang, 2013). The results showed that positive recognition bias for the original items pertaining to profession-related life events in student teachers with strong professional identity was stronger relative to that observed in those with weak professional identity; but this difference was not observed for the additional items pertaining to profession-related life events.

One explanation for this finding could be consistent with cognitive-processing bias theory, which posits that, during cognitive processing, most individuals select information that is consistent with their self-schemas, and this type of selection bias is widespread in attention and memory (Rusting and Larsen, 1998; Heikkilä et al., 2012; Yu et al., 2015). Therefore, relative to those with weak professional identity, student teachers with strong professional identity could have exhibited greater recognition bias for positive items that were consistent with their self-schemas, recognized a higher number of positive original items, and enjoyed obvious processing advantages resulting from positive recognition bias for the original items. Student teachers with strong professional identity paid greater attention to the positive profession-related information, which indicated positive cognitive-processing bias for positive information. However, recognition of additional items pertaining to profession-related life events did not differ significantly between student teachers with strong and weak professional identity. This result could be explained by the findings reported by Wei (2008), who found that, during the learning process, student teachers may have filer and self-explain the professional information consistent with their familiar, showing a consistent with self-cognitive processing. Therefore, familiarity was a vital influential factor, which could have influenced recognition rates.

These results could also be explained by the findings of another study, which showed that professional identity was associated with professional self-schema (Hoeve et al., 2014). Individuals’ professional identity has been shown to influence their professional self-schemas, which affected their cognitive-processing bias (Donald, 2003; DeMeis et al., 2007; Wei et al., 2017). In addition, Markus (1980) posited that individuals could be more likely to select information that is consistent with their professional self-schemas and ignore inconsistent information during learning. In light of these findings, individuals with strong professional identity could exhibit a high degree of sensitivity to stimulation that is consistent with their professional self-schemas. In addition, student teachers with positive professional self-schemas and strong professional identity could pay greater attention to positive information and remember it more easily, relative to other student teachers.

However, the results also showed that student teachers with weak professional identity did not exhibit negative cognitive processing in coding bias for positive items or recognition bias for original items, which was inconsistent with the results of previous research (Wei, 2008). This discrepancy could have occurred because student teachers with weak professional identity were ambivalent toward their occupation. From one perspective, they agreed that teaching was important but had not internalized this as part of their professional identity. From another perspective, they lacked affection for their occupation (Zhao et al., 2011; Fan et al., 2014) because of the lack of benefits and low social status in teaching (Fuller et al., 2013).

## Conclusion and Implications

In conclusion, student teachers’ professional identity influenced their cognitive-processing biases. Specifically, student teachers with strong professional identity exhibited stronger positive coding bias and recognition bias for positive and original items, respectively, relative to that observed in those with weak professional identity. In addition, student teachers with strong professional identity exhibited positive professional self-schemas.

The results of the study identified and distinguished between different cognitive-processing biases for student teachers with strong and weak professional identity. The findings indicated that it would be advisable to provide psychological counseling and cognitive-regulation strategies to improve professional identity, particularly for student teachers with weak professional identity. In addition, universities should use standardized measurement instruments to dissuade student teachers with weak professional identity from entering the teaching profession. In addition, research should be conducted to explore strategies via which to develop student teachers’ professional identity. For example, Alsup (2006) showed that student teachers enhanced their professional identity via engagement in discourse that provoked transformation of their thinking patterns, which allowed them to confront their existing beliefs regarding their professional identity in a formative manner. This result provided strong evidence of the impact of discourse as a powerful tool via which to shape professional identity. The results of the current study are important and of value in the application of strategies via which to improve student teachers’ professional identity and maintain the quality of future teaching.

## Ethics Statement

This study was carried out in accordance with the recommendations of ethics committee of Center for Mental Health Education and Research of Jiangxi Normal University with written informed consent from all subjects. All subjects gave written informed consent in accordance with the Declaration of Helsinki. The protocol was approved by the ethics committee of Center for Mental Health Education and Research of Jiangxi Normal University.

## Author Contributions

X-qW: Design of the study, data collection, data analysis, paper writing and revising. J-cZ: Data analysis, interpretation of data for the work, and paper writing. LL: Data analysis, interpretation of data for the work. X-yC: Paper revising.

## Conflict of Interest Statement

The authors declare that the research was conducted in the absence of any commercial or financial relationships that could be construed as a potential conflict of interest. The reviewer FB and handling Editor declared their shared affiliation, and the handling Editor states that the process nevertheless met the standards of a fair and objective review.
