# Supplementary material for: Cognitive-Processing Bias in Chinese Student Teachers with Strong and Weak Professional Identity
Source: Front Psychol. 2017 May 15;8:784. doi: 10.3389/fpsyg.2017.00784 (PMC5430070; doi:10.3389/fpsyg.2017.00784)
Supplement: Supplementary file 1 [file Presentation_1.PDF]

Appendixes

Appendix A: Experimental reading material and experimental encoding materials

The Story of Two Colleagues

You are a teacher of a middle school, and you have two collegues who named Zhang and Li. Li is a Chinese teacher( ). He graduated from a normal training college( ). He has been working for many years( ). He is a dependable and experienced teacher. Zhang is a math teacher( ). He graduated from a famous normal university( ). He is a young teacher without too many experiences( ).

On Monday morning, as usual, you all arrived earlier. You started to clean the office. Last week, Zhang was on duty( ). Zhang was outgoing( ), but lacked of personal sanitation( ). The office was full of garbages which Zhang left behind( ). There was a disgusting smell all around the office. When you cleaned the office, Zhang did not realize that his bad habit brought extra labor to you( ). Zhang did not feel sorry for his habit at all( ), which made Li angry( ).

After cleaned the office, Li watered his potted plant( ), and then arranged the books on the desk( ). He signed, "A busy semester will end soon. At the school, I was busy with preparing lessons, having class, correcting students' works and so on. At home, I was busy with doing housework, taking care of children. However, I was less cared about myself( )." Then, he shook his head with a bitter smile( ).

Zhang heard Li's words, speechless for a while( ),and then signed, “I agree with you. I used to dream that I will get some great achievements in my job. However, the

rigid life of teacher was so boring( ). Where is my swinging life ? ”( )

“This job is not easy any more” ( ). After arranging the books, Li lit up a cigarette( ), and breathed out a swirl of cigarette smoke( ), said thoughtfully, "In the beginning, when I just joined in my work, all of my schoolmates and friends told me that the job which I took was the most glorious one in the world( ). It was made contact with a group of naive children( ). I got all of their envies( ). Ha-ha" Li laughed, and then, he shook his head( ), continued his speech( ), " However, now everyone of them was better than me( ). We had a reunion the other day. When I saw my old schoolmates, each one of them were complacent. They were either rich or powerful. Compared with them, I felt especially embarrassed( ). For a long time, I couldn't regulate my mood( )."

After hearing what Li said, Zhang started to complain. He put down the book( ), stepped to Li and said: ' Yeah. We worked hard everyday, but how many wages can we get? ( ) It is important that education is increasingly considered by government, public and students ' parents now. On the one hand, it’s a good thing that we can realize the value of our occupation. But on the other hand, indeed, it brings some negative effects. The more important people value the education , the more strict they require teachers. After it ,coming with various inspections( ). We would be supervised by the government, the educational departments, as well as the parents. We prepare courses, have classes, manage students all day long, which is already a tough task. At the same time, we still have to do any thing possible to deal with the inspections( ). How annoying! Although our country made great policies, some

people just shout them in words or print them on the papers instead of implementing these policies. It puts education in a difficult position. '

"Chap! Pain and happiness originated from heart! "Hearing this, Zhang also complained, Li switched the conversation( ), said, "what you said was right. Nowadays, there may be problem in some respects of our education. But, generally, our education has obtained the unprecedented development in recent years. Compared with past, the treatment for our teachers has been greatly improved( ). If we only paid attention to the dark side and blamed it, there might be no effects. Sometimes even became worse. The only thing we could get from that was the encouragement of the ignorance of others and our own .In contrast, if we save our energy to change ourselves, and to participate in teaching, we could have better results. It's impossible to demand the environment adapt to us. Only our adaption to the environment could gradually change our environment."

Zhang looked up to Li( ), while he just wanted to say something but stopped( ). To be honest, he highly admired Li as he worked in an active attitude in this age( ). Although sometimes he complained about the work, as well as flaunting his seniority to younger teachers, he actually got a bunch of achievements in teaching( ). He was a generous people( ). He was achieved the admiration of students ( ).

Zhang, this is your chances! "At this moment, another colleague pushed into and said, "this semester, the school would recommend Zhang to attend young teachers' public lesson competition around city. It would keep you busy for a while. Your passion could finally have a place to exert. To have a better future, you need to work

more hard.” “Thank you, this is indeed a good news to me( ), I must be prepared well, I will be busy! “Said Zhang were filled with joy” ( ).

In the following days, Zhang devoted himself totally to preparing for the public class( ) .There was no his hearty laughter in the office and no his familiar figure on the basketball court any more. No classe time, he always spent half of the day sitting in front of the computer to search information, preparing lessons and designing PPT. Moreover, in order to solve several technical problems, he often consulted with some people who were proficient in computer. Occasionally, he looked thoughtfully when others came across him at school.

Where there is a will there is a way. Zhang finally got a big reward( ). He won the first prize in young teachers’ public lesson competition around city ( ). He will represent the city to take part in the open class competition in province ( ). After the competition, his passion for life was ignited again! Working for two years, finally ,he found the passion of his life as well as the core of his work! ( )

Appendix B: Experimental recognition materials

Instructions: Dear classmates! According to the materials that you just read, please answer the following 108 questions one by one. If the content of the question matches the reading material, please mark a “√” in the parentheses; if not, please mark a “x”. You will have 15 minutes to finish it.

1. Does all the students like Li? ( )

2. Is Li a Chinese language teacher? ( )

3. Is Zhang outgoing? ( )

4. Does Zhang think the school life is very boring? ( )

5. Does Li open his computer? ( )

6.Does Li like to communicate with students? ( )

7. Is Zhang on duty last week? ( )

8. Does Zhang misplaced garbage in the office? ( )

9. Does Li like watching football game? ( )

10. Does Zhang say his school's treatment is not better than others? ( )

11. Is Li a young teacher without too many experiences? ( )

12. Does Zhang teach physics? ( )

13.Does his classmates and friends say that Li chose the most honored career when he started to work? ( )

14. Is Li a kindhearted person? ( )

15.Does Zhang work overtime at night to prepare for the open course? ( )

16.Does Li shake his head with a bitter smile? ( )

17.Does Zhang feel surprised to what Li sighed? ( )

18.Is Zhang's office desk a mess? ( )

19.Does Li say that he ignored himself unconsciously when he was busy? ( )

20.Does Zhang think his mates communicate little with each other everyday? ( )

21.Does Li is respected by his classmates and friends? ( )

22.Does Li lit up a cigarette after putting away books? ( )

23.Does Li breathed out a swirl of cigarette smoke? ( )

24.Does Li say it is hard to be a teacher? ( )

25.Does Zhang publish the teaching thesis on journals? ( )

26.Does Zhang get generous rewards after working so hard? ( )

27.Does Zhang turn off the computer? ( )

28. Does Li shake his head? ( )

29. Does Li teach history? ( )

30. Does Li graduate from a famous national university? ( )

31. Doesn't Li unwilling to help his colleague? ( )

32.Does Li make a cup of tea? ( )

33. Does Zhang teach math? ( )

34.Does Li say that he ignores his children since he was busy? ( )

35. Does Zhang put down the preparation of textbooks? ( )

36. Is Li generous? ( )

37.Does Zhang have two classes on this morning? ( )

38.Does Li continue to deliver his speech? ( )

39.Does Li quit smoking? ( )

40.Is it true that Zhang didn't realize that his bad habits have added a lot of works to us? ( )

41. Is it true that Zhang didn't show his regret? ( )

42.Does Li work actively? ( )

43.Does Li work carefully? ( )

44.Does Zhang win the second prize in the race of the province's public class? ( )

45.Does Zhang work more steady than before through the public lesson competition? ( )

46.Does Li have been working for years? ( )

47.Does Zhang say that where is the colorful life? ( )

48.Does Li say that the education management departments didn’t make enough allowances for the teachers’ hard work? ( )

49.Was Zhang dull when he heard Li’s words? ( )

50.Does Li water the potted plant on the desk? ( )

51.Does Zhang say that it is a good news for him to attend young teachers public lesson competition? ( )

52. Does Li graduated from a local normal college? ( )

53.Does Li dislike to help new colleagues? ( )

54.Will Zhang represent the city to join in the province's public lesson competition? ( )

55.Does Zhang is named "the advanced worker"? ( )

56.Does Li quit the reunion? ( )

57.Does Zhang have been teaching for five years? ( )

58.Does Zhang pay less attention to personal sanitation? ( )

59.Does Li feel very honored for his career at the beginning? ( )

60.Does Zhang say that a teacher works hard while get a low salary? ( )

61.Does Li switched the conversation when Zhang also started to complains? ( )

62.Does Li graduate from a comprehensive university? ( )

63.Does Li say the teacher's treatment also had greatly improved compared with the past? ( )

64.Does Zhang looked up at Li ? ( )

65.Does all the colleagues like Li? ( )

66. Does Zhang like to play basketball ? ( )

67.Does Li teach well? ( )

68.Does Zhang just work for two years? ( )

69.Was Li wrinkly to knit his brows? ( )

70.Does Li say all of his classmates are better than him? ( )

71.Does Zhang's teaching performance is better than before, after the public lesson? ( )

72.Does Zhang say that teachers should try to deal with various checks? ( )

73.Does Li scolded Zhang do not pay attention to public sanitation? ( )

74.Does Zhang think that the teachers have less spare time? ( )

75.Does Li have vertebral cervical ache? ( )

76.Does Li feel embarrassed when compared with classmate on the reunion? ( )

77.Did Zhang go for a trip last weekend? ( )

78. Does Zhang’s passion for life was lit up again, after the public lesson? ( )

79. Is Li a departmental director ? ( )

80.Does Li say that our education got the unprecedented development? ( )

81. Does Zhang just want to say something but stopped again? ( )

82. Is Zhang very frankness ? ( )

83. Does Zhang say that the teachers need to do a lot of meaningless works? ( )

84. Does Zhang get along better with colleagues, after the public lesson? ( )

85. Does Zhang annoy Li for his less attention to the public sanitation? ( )

86. Does Li sighed as he corrected the homework? ( )

87. Does Li's classmates and friends say that he is getting along with a group of naive children? ( )

88. Does Zhang ask the teacher questions actively? ( )

89. Is Li very satisfied with the last salary? ( )

90. Does Zhang say that now the government, society and parents pay more and more attention to education; on the other hand, it also bring a lot of negative effects to teachers? ( )

91. Did Zhang graduate from a local college? ( )

92. Does Li love to flaunt his seniority to the young teacher? ( )

93. Does Zhang pay less attention to speaking? ( )

94.Is it true that Li did not adjust his mood for a long time, after the reunion? ( )

95.Does Zhang devote himself totally into the public lesson? ( )

96. Does Zhang say that his life was enriched? ( )

97.Does Li say that the teaching conditions of school have been greatly improved than before? ( )

98.Does Zhang break his colleague’s vase? ( )

99.Does Li arrange the books on the desk? ( )

100.Does Li's classmates and friends are very envious of him when he just worked at

that time?( )

101. Does Zhang say that some parents put up with too much excessive demands ? ( )

102.Does Li think his classmates achieved more better than him? ( )

103.Does Zhang say that he wanted to have a good preparation for the young teachers' public lesson competition around city ? ( )

104.Does Zhang like his career after the public lesson? ( )

105.Was Li named the "teaching expert"? ( )

106. Is it true that Li didn't contact with his classmates after the reunion? ( )

107. Does Zhang won the first prize in the public lesson competition around the city? ( )

108. Does Zhang finally find his passion of life and the focus of work after the public lesson? ( )
